# Association of gastrointestinal symptoms and skipping breakfast with anxiety and depressive symptoms in quarantined Chinese college students during the Shanghai 2022 lockdown: a cross sectional survey

**DOI:** 10.1186/s12888-023-05317-3

**Published:** 2023-11-28

**Authors:** Tingting Qiao, Dingwei Gao, Gaixia Lu, Wanwan Yi, Zhongwei Lv

**Affiliations:** Department of Nuclear Medicine, Shanghai Tenth People’s Hospital, Tongji University School of Medicine, No. 301, Middle Yanchang Road, Jing’an District, Shanghai, 200072 China

**Keywords:** Quarantined college students, COVID-19 pandemic, Anxiety symptoms, Depressive symptoms, Gastrointestinal symptoms, Skipping breakfast

## Abstract

**Background:**

This study aimed to evaluate the prevalence of anxiety and depressive symptoms among quarantined college students at school in Shanghai 2022 lockdown during the COVID-19 pandemic and investigate the association of gastrointestinal discomfort related-factors and skipping breakfast with anxiety and depressive symptoms.

**Methods:**

384 quarantined college students in Shanghai China were recruited in this cross-sectional study from April 5th to May 29th, 2022. Generalized Anxiety Disorder (GAD-7) and Patient Health Questionnaire (PHQ-9) were used to assess anxiety and depressive symptoms, respectively.

**Results:**

The prevalence of anxiety and depressive symptoms were 56.8% and 62.8%, respectively. Longer quarantine duration, higher education level, skipping breakfast, stomachache or abdominal pain, and nausea or dyspepsia were significantly associated with anxiety symptoms. Moreover, longer quarantine duration, being woman, skipping breakfast, stomachache or abdominal pain, and nausea or dyspepsia were markedly related to depressive symptoms. Notably, regularly physical exercising and taking positive attitude towards COVID-19 were negatively correlated with anxiety and depressive symptoms.

**Conclusions:**

More attention should be paid to anxiety and depressive symptoms of quarantined college students and universities should provide timely psychological monitoring and intervention services to mitigate the impact of negative emotions on students. Effectively relieving gastrointestinal symptoms, insisting on eat breakfast, regularly exercising, and taking a positive attitude towards to COVID-19 might contribute to preventing the anxiety and depressive symptoms for those college students experiencing a long-term quarantine.

## Introduction

Coronavirus disease (COVID-19), the infection of novel coronavirus, is a serious pneumonia pandemic and has broken out all over the world [1]. From March to June 2022, Shanghai, the largest city in China, experienced the largest clustered infection of COVID-19 since 2020. To eradicate the spread of COVID-19, 24.9 million population in Shanghai were therefore experienced an unprecedented strict lockdown and mass testing [2]. Such a large-scale lockdown in Shanghai is the largest known city-wide lockdown globally, providing a unique background different from other Chinese cities and elsewhere in the world for assessing anxiety and depressive symptoms caused by the large-scale city-wide lockdown.

During the city-wide lockdown period, all population of Shanghai have been issued stay-at-home orders, including restricting the flow of people and self-quarantine at home, etc. [2]. Strict self-quarantine, preventing exposure to a COVID-19 positive individual, is an effective measure for quickly containing the pandemic [3]. Although self-quarantine could reduce the spread of COVID-19, quarantine may lead to adversely impact on public mental health [4], especially for those prone to gastrointestinal discomfort [5, 6]. Gastrointestinal discomfort symptoms, such as stomachache, abdominal pain, nausea, dyspepsia, constipation and diarrhea, are a series of common symptoms that may occur in the general population, and people with these symptoms are more likely to experience more personal pressure and further develop into psychological problems. Many investigations have confirmed that gastrointestinal symptoms are closely related to psychological distress [7–12]. In addition, there are many studies currently focusing on the adverse effects of skipping breakfast on the physical health. For example, previous studies demonstrated that skipping breakfast was associated with prediabetes [13] and gynecologic disorders [14], etc. Interestingly, skipping breakfast has also been reported to be associated with negative emotions [15, 16]. Previous studies showed that skipping breakfast was positively correlated with psychological distress [17–19]. However, few studies have focused on the relationship between skipping breakfast and psychological problems under the influence of COVID-19 pandemic.

Recent studies have demonstrated that the incidence of psychological problems increased in the general population during the COVID-19 pandemic [20–25]. Self-quarantine further aggravates the occurrence of psychological disorders [26–33]. A previous study showed that the population quarantined for two week had higher levels of depression, anxiety and insomnia symptoms than the general population [27]. Another study demonstrated that longer quarantine duration was associated with increased social distancing and psychological distress in Chinese adults during the outbreak of COVID-19 [34]. Moreover, long-term self-quarantine measures are associated with an increased risk of physical discomfort. Sun et al. showed that long-term quarantine increased the risk of experiencing pain, and psychological symptoms could mediate the impact of the quarantine on pain symptoms [35]. Nakov et al. reported that the prevalence of gastrointestinal symptoms significantly increased during the COVID-19 lockdown and may was associated increased numbers of patients with disorder of brain-gut interaction [5].

Previous studies have shown that college students were vulnerable to mental problems [36–38] and suffer from more negative emotions during the COVID-19 pandemic [39] than before the pandemic [40]. Gao et al. documented the prevalence of anxiety and depression were 32.4% and 46.4% among college students during the COVID-19 outbreak [39]. Most of the research on college students’ psychological problems was carried out in the normalization stage of the COVID-19 pandemic [41, 42]. In addition, a few studies have observed the anxiety and depression levels of Chinese college students who were isolated at home with the closure of schools after the initial outbreak of COVID-19 [43]. However, to our best knowledge, few previous studies have investigated the prevalence of anxiety and depressive symptoms among college students who were quarantined at school during the lockdown of the COVID-19, especially during the period in 2022 when the second outbreak of COVID-19 led to a city-wide lockdown in Shanghai. In Shanghai city-lockdown period, colleges students experienced unprecedented strict management policies, such as restricting the flow of students without permission, implementing the “online teaching” mode, requiring all students to have COVID-19 testing every day, and sending some students at risk to centralized quarantine. This likely increased their negative emotions for containment restrictions, adverse information, and fear for infection of COVID-19, etc. Therefore, quarantined college students at school should be paid more attention to their mental health and related influencing factors during the city-wide lockdown period, thereby promptly detect psychological problems and conduct psychological intervention.

The purpose of this study was to determine the prevalence of anxiety and depressive symptoms in college students quarantined at school in Shanghai 2022 lockdown during COVID-19 pandemic by using self-reported questionnaires, and to explore the association of gastrointestinal symptoms and skipping breakfast with anxiety and depressive symptoms.

## Materials and methods

### Participants

This online cross-sectional study was conducted from April 5th to May 29th, 2022, the period of the second outbreak of COVID-19 in Shanghai, China, and college students were quarantined at school under strictly state-enforced lockdown. During this period, quarantined college students were invited to participate in the current anonymous online survey through the Wenjuanxing platform (Changsha Haoxing Information Technology Co., Ltd., China), which can be accessed by scanning the QR code or clicking the link on WeChat. The first page of the questionnaire shows informed consent, so those who completed the questionnaire voluntarily participated in the study. The study was approved by the ethics committee of the Hubei Maternal and Child Health Hospital (#FYGG(L)2020–004), complied with the Declaration of Helsinki.

In total, this electronic questionnaire was clicked 418 times. The inclusion criteria were quarantined college students at school, no restrictions on majors. These participants were excluded: (a) those who were not being quarantined, or not college students, or gave incomplete responses; and (b) those who had any chronic medical disease or with a known psychiatric illness. Finally, a total of 384 participants were included and analyzed, with an effective response rate of 91.87%.

### Measurements and covariates

The questionnaire consists of the following parts: (a) purpose of this study and informed consent; (b) basic information questionnaire including age, height, weight, quarantine duration, marital status (unmarried and married), education level (undergraduate, postgraduate, and doctoral student), skipping breakfast, physical exercise, and attitude towards COVID-19; (c) Generalized Anxiety Disorder (GAD-7) and Patient Health Questionnaire (PHQ-9); (d) gastrointestinal discomfort symptoms including stomachache or abdominal pain, nausea or dyspepsia, and constipation or diarrhea.

Quarantine duration was measured by the questions: “How long have you been quarantined?” Regular physical exercise was defined as participants who answer “Yes” of the question of “Do you exercise regularly exceeded three times a week?”. Positive attitude towards COVID-19 was measured by the questions: “Do you have a positive attitude towards COVID-19 pandemic?”. The participants’ self-reported “Yes” were considered as positive attitude towards COVID-19. Skipping breakfast was measured by the questions: “Did you ever skip breakfast in the past week?”. This is a three-point Likert-type scale from 0 “rarely” 1 “sometimes” to 2 “often”. We divided “sometimes” and “often” into skipping breakfast group according to the answer of participants. In addition, the symptoms of stomachache or abdominal pain was measured by the questions: “Have you had stomachache or abdominal pain in the past week?”, and the potions included “no pain at all”, “seldom” and “often”. The participants self-reported “no pain at all” or “seldom” were defined as the no-pain group, while “often” was considered as the stomachache or abdominal pain group. Also, nausea or dyspepsia was assessed by the questions: “Have you had nausea or dyspepsia in the past week?” with the options of “never”, “seldom” and “often”. We defined “often” reported by participants as nausea or dyspepsia group. And constipation or diarrhea was assessed by the questions: “Have you had constipation or diarrhea in the past week?” with the options of “Yes” or “No”. Constipation was defined as defecating less than three times a week, and diarrhea was defined as defecating more than three times a day. The weight divided by the square of height was used to calculate body mass index (BMI).

### Anxiety and depression

GAD-7 [44], a seven-item self-assessment scale, was conducted to evaluate anxiety symptoms. The Chinese versions of GAD-7 has been proved to be an effective method to measure the symptoms of anxiety among Chinese population [45–47], with a recommended cutoff score of 5 or more. Depressive symptoms were assessed by PHQ-9 [48], which is composed of 9 items and has been widely performed to assess the depression level of Chinese people [49–51]. Depressive symptoms are defined as the score of 5 or more.

### Data analysis

We used medians and interquartile ranges (IQRs) to present continuous variables and proportions (%) to present categorical variables. Continuous variables were compared using Mann–Whitney U test, and Chi-square test were conducted to analyze categorical variables. Bonferroni adjustment was carried out for multiple comparisons. In addition, binary logistic regression analysis presented as odds ratio (OR) and 95% confidence intervals (95% CI) was applied to examine which factors were independently associated with anxiety and depressive symptoms. Moreover, confounding variables including age, sex, quarantine duration, marital status, education level, BMI, skipping breakfast, regular physical exercise, positive attitude towards COVID-19, stomachache or abdominal pain, nausea or dyspepsia, and constipation or diarrhea were controlled to adjust logistic regression analysis. The analyses were performed with SPSS Statistics 26 (IBM Corporation, Armonk, NY, USA) and statistical significance was set at *p* < 0.05.

## Results

### Basic characteristics and prevalence of anxiety and depressive symptoms

Detailed basic characteristics are shown in Table 1. A total of 384 quarantined college students at school in Shanghai lockdown were enrolled in this study. The prevalence of anxiety and depressive symptoms in participants were 56.8% and 62.8%, respectively. The median (IQR) age of participants was 25(24.0–27.0) years, and the median (IQR) quarantine duration was 50(40.0–60.0) days. Majority of participants were woman (69.3%), unmarried (88.0%), postgraduate (49.6%), skipping breakfast (69.5%), less regular exercise (54.7%), and had a positive attitude towards COVID-19 (51.0%). Moreover, 33.9%, 41.1%, and 39.3% of participants suffered stomachache or abdominal pain, nausea or dyspepsia, and constipation or diarrhea, respectively.

**Table 1:** Characteristics of quarantined college students by anxiety and depression status

			GAD-7				PHQ-9			
Variables	Category	Total	Non-anxiety	Anxiety	*P* value	Adjusted *p*-value^a^	Non-depression	Depression	*P* value	Adjusted *p*-value^a^
Total (n, %)		384	166(42.2)	218(56.8)			143(37.2)	241(62.8)		
Age (years), [M (IQR)]		25(24.0–27.0)	25(24.0–27.0)	25(23.0–27.0)	0.713	> 0.999	25(24.0–27.0)	25(23.5–27.0)	0.343	0.686
Sex (n, %)					0.119	0.238			0.210	0.420
	Man	118(30.7)	58(49.2)	108(50.8)			54(45.8)	64(54.2)		
	Woman	266(69.3)	108(40.6)	158(59.4)			89(54.2)	177(66.5)		
Quarantine duration (days) [M (IQR)]		50(40.0–60.0)	45(36.8–60.0)	55(40.8–60.0)	< 0.001	< 0.001	41(35.0–60.0)	56(40.0–60.0)	< 0.001	< 0.001
Marital status (n, %)					0.779	> 0.999			0.489	0.978
	Unmarried	338(88.0)	147(43.5)	191(56.5)			128(37.9)	210(62.1)		
	Married	46(12.0)	19(41.3)	27(58.3)			15(32.6)	31(67.4)		
Education level (n, %)					0.072	0.114			0.448	0.896
	Undergraduate	60(15.6)	34(56.7)	26(43.7)			23(38.3)	37(61.7)		
	Postgraduate	190(49.6)	78(41.1)	112(58.9)			65(34.2)	125(65.8)		
	Doctoral student	134(34.9)	54(40.3)	80(58.7)			55(41.0)	79(59.0)		
BMI [M (IQR)]		20.76(19.3–22.8)	20.76(19.2–22.8)	20.76(19.5–22.8)	0.855	> 0.999	20.96(19.3–22.9)	20.58(19.5–22.7)	0.413	0.826
Skipping breakfast (n, %)					0.003	0.006			0.031	0.062
	No	117(30.5)	64(54.7)	53(45.3)			53(45.3)	64(54.7)		
	Yes	267(69.5)	102(38.2)	165(61.8)			90(33.7)	177(66.3)		
Regular physical exercise (n, %)					0.430	0.860			0.127	0.254
	No	210(54.7)	81(38.6)	129(61.4)			71(33.8)	139(66.2)		
	Yes	174(45.3)	85(48.9)	89(51.1)			72(41.4)	102(58.6)		
Positive attitude towards COVID-19 (n, %)					0.500	> 0.999			0.525	> 0.999
	No	188(49.0)	78(49.0)	110(58.5)			67(35.6)	121(64.4)		
	Yes	196(51.0)	88(51.0)	108(55.1)			76(38.8)	120(61.2)		
Stomachache or abdominal pain (n, %)					0.150	0.300			< 0.001	< 0.001
	No	254(66.1)	121 (47.6)	133(52.4)			111(43.7)	143(56.3)		
	Yes	130(33.9)	45(34.6)	85(65.4)			32(24.6)	98(75.4)		
Nausea or dyspepsia (n, %)					0.310	0.620			0.011	0.022
	No	226(58.9)	108(47.8)	118(52.2)			96(42.5)	130(57.5)		
	Yes	158(41.1)	118(36.7)	100(63.3)			47(29.7)	111(70.3)		
Constipation or diarrhea (n, %)					0.780	> 0.999			0.630	> 0.999
	No	233(60.7)	141(43.8)	108(56.2)			89(38.2)	114(61.8)		
	Yes	151(39.3)	68(42.4)	49(57.6)			54(35.8)	97(64.2)		

*Abbreviations*: *GAD-7* Generalized Anxiety Disorder Questionnaire, *PHQ-9* Patient Health Questionnaire, *BMI* Body mass index, *IQR* Interquartile range. Adjusted *p*-value^a^ was obtained from Bonferroni adjustments

Compared with the non-anxiety group, the anxiety group had longer quarantine duration (45 days vs. 55 days, *p* < 0.001), and more skipping breakfast (38.2% vs. 61.8%, *p* = 0.003). In addition, compared with the non-depression group, the depression group had longer quarantine duration (41 days vs. 56 days, *p* < 0.001), more skipping breakfast (33.7% vs. 66.3%, *p* = 0.031), more had stomachache or abdominal pain (24.6% vs. 75.4%, *p* < 0.001), and more had nausea or dyspepsia (29.7% vs. 70.3%, *p* = 0.011). However, after Bonferroni adjustments, there was no significant difference in skipping breakfast between the non-depression group and the depression group.

### Factors associated with the symptoms of anxiety and depression

Multivariate logistic regression (Table 2) showed that quarantine duration (OR, 1.039, 95% CI, 1.021–1.057), skipping breakfast (OR, 2.329, 95% CI, 1.439–3.770), stomachache or abdominal pain (OR, 2.071, 95% CI, 1.135–3.780), and nausea or dyspepsia (OR, 1.810, 95% CI, 1.009–3.249) were significantly associated with anxiety symptoms among quarantined college students. Moreover, postgraduate (OR, 2.304, 95% CI, 1.188–4.467) and doctoral student (OR, 2.511, 95% CI, 1.257–5.013) showed a higher odds of anxiety symptoms compared to undergraduate. And regular physical exercise (OR, 0.579, 95% CI, 0.370–0.904) and positive attitude towards COVID-19 (OR, 0.432, 95% CI, 0.245–0.763) were less likely show the anxiety symptoms.

**Table 2:** Univariate and multivariate analyses of the anxiety symptoms among the quarantined college students

Variables	Category		Unadjusted OR (95% CI)	*P* value	Adjusted OR (95% CI)	*P* value
Age			0.997(0.937–1.061)	0.932	1.018(0.948–1.092)	0.631
Sex	Man	Reference				
	Woman		1.414(0.914–2.187)	0.119	0.613(0.356–1.055)	0.077
Quarantine duration			1.036(1.020–1.052)	< 0.001	1.039(1.021–1.057)	< 0.001
Marital status	Unmarried	Reference				
	Married		1.094(0.585–2.043)	0.779	0.989(0.493–1.984)	0.976
Education level	Undergraduate	Reference				
	Postgraduate		1.878(1.044–3.376)	0.035	2.304(1.188–4.467)	0.014
	Doctoral student		1.937(1.046–3.588)	0.035	2.511(1.257–5.013)	0.009
BMI			0.995(0.926–1.069)	0.890	1.020(0.932–1.117)	0.663
Skipping breakfast	No	Reference				
	Yes		1.953(1.258–3.033)	0.003	2.329(1.439–3.770)	0.001
Regular physical exercise	No	Reference				
	Yes		0.657(0.438–0.988)	0.043	0.579(0.370–0.904)	0.017
Positive attitude towards COVID-19						
	No	Reference				
	Yes		0.870(0.581–1.304)	0.500	0.432(0.245–0.763)	0.004
Stomachache or abdominal pain	No	Reference				
	Yes		1.718(1.110–2.661)	0.015	2.071(1.135–3.780)	0.018
Nausea or dyspepsia	No	Reference				
	Yes		1.578(1.041–2.391)	0.031	1.810(1.009–3.249)	0.047
Constipation or diarrhea	No	Reference				
	Yes		1.058(0.700–1.601)	0.788	0.899(0.534–1.514)	0.689

*Abbreviations*: *OR* Odds ratio, *CI* Confidential interval, *BMI* Body mass index

Confounding variables include: age, sex (man/woman), quarantine duration, marital status (unmarried/married), education level (undergraduate/ postgraduate/ doctoral student), BMI, skipping breakfast(yes/no), regular physical exercise(yes/no), positive attitude towards COVID-19(yes/no), stomachache or abdominal pain(yes/no), nausea or dyspepsia(yes/no), and constipation or diarrhea(yes/no)

In the multivariate logistic regression analysis (Table 3), compared with male college students, female college students were associated with increased odds of depressive symptoms (OR, 1.800, 95% CI: 1.037–3.123). In addition, quarantine duration (OR, 1.039, 95% CI, 1.021–1.057), skipping breakfast (OR, 1.945, 95% CI, 1.187–3.186), stomachache or abdominal pain (OR, 3.136, 95% CI, 1.656–5.941), and nausea or dyspepsia (OR, 1.892, 95% CI, 1.032–3.470) were also significantly associated with depressive symptoms. Also, regular physical exercise (OR, 0.607, 95% CI, 0.382–0.964) and positive attitude towards COVID-19 (OR, 0.380, 95% CI, 0.212–0.682) were negatively correlated with depressive symptoms.

**Table 3:** Univariate and multivariate analyses of the depressive symptoms among the quarantined college students

Variables	Category		Unadjusted OR (95% CI)	*P* value	Adjusted OR (95% CI)	*P* value
Age			0.956(0.897–1.019)	0.167	0.964(0.897–1.036)	0.323
Sex	Man	Reference				
	Woman		1.678(1.078–2.613)	0.022	1.800(1.037–3.123)	0.037
Quarantine duration			1.038(1.022–1.055)	< 0.001	1.039(1.021–1.057)	< 0.001
Marital status	Unmarried	Reference				
	Married		1.260(0.655–2.424)	0.489	1.206(0.582–2.496)	0.615
Education level	Undergraduate	Reference				
	Postgraduate		1.195(0.656–2.179)	0.560	1.334(0.674–2.638)	0.408
	Doctoral student		0.893(0.478–1.666)	0.722	0.995(0.493–2.009)	0.989
BMI			0.978(0.908–1.052)	0.547	1.037(0.945–1.139)	0.445
Skipping breakfast	No	Reference				
	Yes		1.629(1.045–2.538)	0.031	1.945(1.187–3.186)	0.008
Regular physical exercise	No	Reference				
	Yes		0.724(0.478–1.097)	0.127	0.607(0.382–0.964)	0.034
Positive attitude towards COVID-19	No	Reference				
	Yes		0.874(0.578–1.323)	0.525	0.380(0.212–0.682)	0.001
Stomachache or abdominal pain	No	Reference				
	Yes		2.377(1.486–3.803)	< 0.001	3.136(1.656–5.941)	< 0.001
Nausea or dyspepsia	No	Reference				
	Yes		1.744(1.133–2.684)	0.011	1.892(1.032–3.470)	0.039
Constipation or diarrhea	No	Reference				
	Yes		1.110(0.726–1.698)	0.630	0.791(0.461–1.359)	0.396

*Abbreviations*: *OR* Odds ratio; *CI* Confidential interval, *BMI* Body mass index

Confounding variables include: age, sex (man/woman), quarantine duration, marital status (unmarried/married), education level (undergraduate/ postgraduate/ doctoral student), BMI, skipping breakfast(yes/no), regular physical exercise(yes/no), positive attitude towards COVID-19(yes/no), stomachache or abdominal pain(yes/no), nausea or dyspepsia(yes/no), and constipation or diarrhea(yes/no)

In this study, age, marital status, BMI, and constipation or diarrhea were no significant statistical relationships with anxiety and depressive symptoms (*p* > 0.05).

## Discussion

Self-quarantine is one of the most common effective measures to prevent or minimize the spread of COVID-19 among communities and is also one of the strict control measures implemented by the government during the 2022 Shanghai city-wide lockdown. Shanghai universities have implemented a series of measures to prevent college students from infecting COVID-19 during lockdown periods. The most important measure is to require college students to be quarantined in dormitories to strictly restrict student mobility. College students are vulnerable to psychological problems during the COVID-19 pandemic. As a result of the long self-quarantine, college students may experience a higher mental health burden. Therefore, it is of great practical significance to pay attention to the psychological distress of quarantined college students at school during the Shanghai 2022 lockdown.

This study is a cross-sectional survey involving quarantined Chinese college students at school during the height of the unprecedented Shanghai lockdown. To the best of our knowledge, this is the first study to investigate the relationship between gastrointestinal symptoms, skipping breakfast and anxiety and depressive symptoms among quarantined Chinese college students at school during COVID-19 pandemic. We found that the prevalence of anxiety and depressive symptoms among these quarantined college students was 56.8% and 62.8%, respectively. Moreover, longer quarantine duration, skipping breakfast, stomachache or abdominal pain, and nausea or dyspepsia were significantly associated with anxiety and depressive symptoms, while regular physical exercise and positive attitude towards COVID-19 were negatively correlated with anxiety and depressive symptoms. In addition, higher education level was only related to anxiety symptoms, while female gender was only connected with depressive symptoms.

In the present study, the overall prevalence of anxiety and depressive symptoms among survey respondents was 56.8% and 62.8%. The proportions of anxiety and depressive symptoms of this study were relatively high compared with other quarantined general population in China. For example, in one study of 1214 participates quarantined only once in early 2020 in Chengdu reported the prevalence of depressive symptoms was 34.2% [26]. In another study showed that the prevalence of generalized anxiety symptoms was 37.69% among the 10,824 individuals in China [52]. Li et al., showed that the prevalence of anxiety and depressive symptoms were 7.26 and 16.11% among discharged COVID-19 patients in Wuhan during the home isolation period [53]. The high prevalence of depressive and anxiety symptoms in quarantined college students may be related to the severity of the COVID-19 pandemic. The 2022 Shanghai city-wide lockdown was the largest known city-wide lockdown in the world, thus the strict control measures implemented by the government and the panic for COVID-19 infection probably aggravate their mental burden [54]. Moreover, the quarantine duration [50 (40.0–60.0) days] of these college students was much longer than participants who were usually only quarantined for one or two weeks in other investigations [26, 35]. Additionally, our research findings believed that quarantined college students may be vulnerable to anxiety and depressive symptoms than other groups.

Rates of mental health problems among Chinese college students during the normalization stage of COVID-19 pandemic were reported ranging from 7.3 to 41.4% for anxiety and 21.2 to 46.4% for depression. Zhan et al., reported that 43.77% of college students showed depressive symptoms during the COVID-19 pandemic normalization in China [49]. During the COVID-19 outbreak period, Gao et al. found 32.4% and 46.4% of Chinese college students showed anxiety and depressive symptoms [39]. Compared to studies conducted in a normal period, a relatively higher prevalence of anxiety and depressive symptoms was showed in this study. Notably, our data was similar to several survey results of quarantined college students in Jiangsu, China [43] (48.1% for anxiety and 57.6% for depression) and Wuhan [55] (61.64% for anxiety), indicating the importance of paying attention to the mental health of quarantined college students during the COVID-19 lockdown. Altogether, this study indicated widespread mental health problems among long-term quarantined college students at school in Shanghai 2022 lockdown.

Furthermore, we identified several influencing factors for anxiety and depressive symptoms in quarantined college students during Shanghai lockdown. In this study, we found that 69.5% of quarantined college students during the Shanghai lockdown period were skipping breakfast. We speculated that the high rate of skipping breakfast may be related to wide-spread food insecurity caused by the large-scale city-wide lockdown. Quarantined college students skipped breakfast because they cannot meet their personal dietary needs and food preferences. However, skipping breakfast are known to increase the risk of various physical diseases, such as chronic kidney disease [56] and diabetes [57]. Also, previous studies showed that skipping breakfast showed stronger associations with the psychological problems in adolescents [15, 17], but the COVID-19 pandemic was not involved in these investigations. Our study firstly observed that skipping breakfast was closely associated with anxiety and depressive symptoms among quarantined college students during the COVID-19 pandemic lockdown. In note, depressive symptoms (such as insomnia, delayed awakening, or reduced appetite) may influence the habit of skipping breakfast, and to some extent, interactions must be bidirectional. Unfortunately, due to the cross-sectional design of this study, it is not possible to determine the direction of causality in the relationship.

In addition, our findings indicated that longer quarantine duration was significantly related to anxiety and depressive symptoms, which was consistent with previous study that people with longer quarantine duration in China showed increased psychological distress during the COVID-19 pandemic [30]. Besides, we found that female college students were more likely to have depressive symptoms than male college students, which was in accordance with previous research on neuropsychology trainees during the COVID-19 pandemic [58]. In addition, our multivariate logistic analyses showed that postgraduates and doctoral students showed a higher odds of anxiety symptoms than undergraduates respectively, which was confirmed in a previous study of Chinese students [59]. In this study, the findings indicated that regular physical exercise and positive attitude towards COVID-19 were negatively correlated with anxiety and depressive symptoms, which was also consistent with previous studies. A cross-sectional study in Brazilians during the COVID-19 pandemic found people who was not involved with physical exercise had higher level of anxiety and depression [60]. Han et al. found that the weakening of physical exercise of Chinese college students had a negative impact on their emotions in the period of the COVID-19 pandemic [61]. Consistent with our research, a previous research showed that anxious college students believe that COVID-19 has a greater impact on life and had higher levels of concern about COVID-19 [62], that is, they did not maintain a positive attitude towards COVID-19. Overall, these results of the study could offer some useful references for preventing psychological distress problems of quarantined college students during the COVID-19 lockdown.

Interestingly, Oshima et al. demonstrated that the COVID‐19 pandemic negatively affected the gastrointestinal and psychological symptoms in functional dyspepsia and irritable bowel syndrome subjects [63]. Our findings showed that the prevalence of stomachache or abdominal pain, nausea or dyspepsia and constipation or diarrhea among quarantined college students was 33.9%, 41.1%, and 39.3% respectively, but lower than one previous study that gastrointestinal symptoms were reported by 68.9% during the COVID- 19 lockdown [5]. This may be because these participants (age, 40.5 ± 12.6 years) are significantly older than these young college students of our study. There was a pre-COVID-19 study reported that experiencing gastrointestinal problems of Spanish population increased the probability of anxiety and depression, which was consistent with this study [64]. Oliviero et al. observed that higher anxiety level was a risk factor for worsening epigastric burning and abdominal pain in Southern Italy during COVID- 19 lockdown [6]. This study was the first to show that stomachache or abdominal pain and nausea or dyspepsia were significantly associated with anxiety and depressive symptoms in quarantined Chinese college students during the COVID-19 pandemic lockdown, indicating that gastrointestinal symptoms may interact with anxiety and depressive symptoms during the COVID-19 pandemic. We should pay more attention to gastrointestinal symptoms of quarantined college students, especially those who experience stomach pain or abdominal pain and nausea or indigestion.

The main limitations of this study should be noted. This is a cross-sectional design study, which did not explore the causal relationship between psychological symptoms and related research factors. Next, in this study, no general population without isolation was used as the control group. In addition, we only used the single scale (GAD-7 and PHQ-9) to assess anxiety and depressive symptoms. The results of psychological symptoms will be more reliable if multiple recognized effective psychological scales are used to evaluate anxiety and depressive symptoms at the same time. And the positive attitude towards COVID-19 was not measured by the validated scale. The lack of objective measurement of somatic symptoms and clinical interviews with mental distress was also a limitation of this study. Finally, many other factors may also be related to the symptoms of anxiety and depression (such as diet, sleep, parents’ education level, family economy, smoke, and drink). The present research was a cross-sectional design study hence this study may only consider limited factors associated with psychological symptoms and gastrointestinal symptoms during COVID-19 pandemic.

## Conclusion

Our results showed that the prevalence of anxiety and depressive symptoms in the quarantined college students was increased during the 2022 Shanghai lockdown. Although our study is a cross-sectional design study, the findings suggest that more attention should be paid to anxiety and depressive symptoms for quarantined college students, especially those students who experiencing longer quarantine duration, being female students, higher education levels, skipping breakfast, having stomachache or abdominal pain, and having nausea or dyspepsia. At the same time, appropriate and timely psychological intervention should be carried out for quarantined college students with psychological problems to reduce the psychological damage caused by the COVID-19 pandemic. More importantly, colleges should strengthen the psychological skills training for all quarantined college students to better regulate the psychological state and alleviate the psychological distress.

## Data Availability

The data that support the findings of this study are available from the corresponding author, YWW, upon reasonable request, but restrictions apply to the availability of these data, which were used under license for the current study, and so are not publicly available.

## Abbreviations

COVID-19: Coronavirus disease
GAD-7: Generalized Anxiety Disorder
PHQ-9: Patient Health Questionnaire
IQR: Interquartile ranges
BMI: Body mass index
OR: Odds ratio
CI: Confidence interval
